# Tandem Mass Spectrometry de novo Sequencing of the Skin Defense Peptides of the Central Slovenian Agile Frog *Rana dalmatina*

**DOI:** 10.3390/molecules28207118

**Published:** 2023-10-16

**Authors:** Tatiana Yu. Samgina, Irina D. Vasileva, Polonca Trebše, Gregor Torkar, Alexey K. Surin, Zhaowei Meng, Roman A. Zubarev, Albert T. Lebedev

**Affiliations:** 1 Department of Materials Science, MSU-BIT University, Shenzhen 517182, China; 2 Department of Organic Chemistry, Lomonosov Moscow State University, 119991 Moscow, Russia; idvasilieva@gmail.com; 3 Faculty of Health Sciences, University of Ljubljana Zdravstvena Pot 5, 1000 Ljubljana, Slovenia; polonca.trebse@zf.uni-lj.si; 4 Department for Biology, Chemistry and Home Economics, University of Ljubljana Faculty of Education, Kardeljeva Ploščad 16, 1000 Ljubljana, Slovenia; gregor.torkar@pef.uni-lj.si; 5 Pushchino Branch, Shemyakin–Ovchinnikov Institute of Bioorganic Chemistry, Russian Academy of Sciences, Prospekt Nauki 6, Pushchino, 142290 Moscow, Russia; alan@vega.protres.ru; 6 Department of Medicinal Biochemistry and Biophysics, Division of Molecular Biometry, Karolinska Institutet, SE-171 77 Stockholm, Sweden; zhaowei.meng@ki.se (Z.M.); roman.zubarev@ki.se (R.A.Z.); 7 The National Medical Research Center for Endocrinology, 115478 Moscow, Russia; 8 Department of Pharmacological & Technological Chemistry, I.M. Sechenov First Moscow State Medical University, 119146 Moscow, Russia

**Keywords:** agile brown frog, membrane-active peptides, tandem mass spectrometry, top-down de novo sequencing, bradykinin-related peptides (BRPs), EThcD

## Abstract

Peptides released on frogs’ skin in a stress situation represent their only weapon against micro-organisms and predators. Every species and even population of frog possesses its own peptidome being appropriate for their habitat. Skin peptides are considered potential pharmaceuticals, while the whole peptidome may be treated as a taxonomic characteristic of each particular population. Continuing the studies on frog peptides, here we report the peptidome composition of the Central Slovenian agile frog *Rana dalmatina* population. The detection and top-down de novo sequencing of the corresponding peptides was conducted exclusively by tandem mass spectrometry without using any chemical derivatization procedures. Collision-induced dissociation (CID), higher energy collision-induced dissociation (HCD), electron transfer dissociation (ETD) and combined MS^3^ method EThcD with stepwise increase of HCD energy were used for that purpose. MS/MS revealed the whole sequence of the detected peptides including differentiation between isomeric Leu/Ile, and the sequence portion hidden in the disulfide cycle. The array of the discovered peptide families (brevinins 1 and 2, melittin-related peptides (MRPs), temporins and bradykinin-related peptides (BRPs)) is quite similar to that of *R*. *temporaria.* Since the genome of this frog remains unknown, the obtained results were compared with the recently published transcriptome of *R*. *dalmatina*.

## 1. Introduction

The amphibia of the Ranidae family, as well as other anurans, have developed in the process of evolution a unique capability to synthesize and store in their bodies certain peptides [1,2,3]. These peptides may protect them from pathogens and predators [4,5]. They consist of a highly conserved (signal) N-terminal peptide, moderately conserved proregion (acidic spacer) and highly variable C-terminal fragment (mature peptide) [6,7,8,9,10]. When frogs are under stress, certain protease cut the signal peptide and frogs release propeptides onto their skin, while a co-secreted protease liberates mature bioactive peptides [6]. The latter are usually cationic, hydrophobic and have a structure of amphipatic α-spirals. They can actively interact with the membranes of pathogenic cells [6,8]. Being powerful cytolitics, they can also immediately penetrate through the cells’ membranes of the oral cavities of predators, delivering secreted neuropeptides to the targeted neuroreceptors of predators, triggering pain or provoking a vomiting reflex [11,12]. The unique mechanism of the interaction of these peptides with pathogens’ membranes [13,14,15,16,17] as well as their structure–activity relations [13] constitute a platform for the creation of new peptide or peptidomimetic pharmaceuticals capable of fighting highly resistant pathogens without a risk of developing resistance [14,18]. Anyway, the first stage of the frogs’ peptides study involves establishing their sequences. This task is especially challenging as the genomes of the vast majority of frogs remain unknown.

Nowadays, high resolution mass spectrometry is the most powerful tool to sequence peptides [19,20,21]. Top-down de novo sequencing requires accurate mass measurements and the application of complementary tandem mass spectrometry methods to trigger fragmentation. Fourier-transform ion-cyclotron resonance or various orbital traps are the most efficient for this purpose [19]. Liquid chromatography with prolonged elution times is also quite helpful to define the peptidome of each new species, including the minor components. The applied option of the dynamic exclusion of the most intense peptide molecular ions enables recording tandem mass spectra of these minor components of the skin secretion almost simultaneously with the major constituents included in the dynamic exclusion list. The latter approach gives a chance to detect the smallest changes in the composition of skin peptidomes, which is highly important for the studies of differences between populations of the same species [22]. Visualization of the peptidome composition using 2D maps proved its usefulness for the differentiation of frog species and populations as well as its ability to predict the possible types of bioactivity of novel peptides [23]. So far, three methods of tandem mass spectrometry demonstrate the highest efficiency in these studies: collision-induced dissociation (CID), higher energy collision-induced dissociation (HCD), and combined MS^3^ method EThcD with stepwise increase of HCD energy [24]. Although novel algorithms of sequencing become more and more useful (see Results and Discussion), manual spectra interpretation remains the most reliable tool so far [25]. Thus, the completeness of establishing the peptidome depends on the accuracy of mass measurements, rate of spectra acquisition, available tandem mass spectrometry methods, chromatography parameters, details of the sample preparation, as well as the experience of the spectra investigator. Correctly addressing these issues enables detecting even the least abundant components of each peptidome.

cDNA cloning as an alternative tool to establish peptidomes provides the corresponding transcriptome. It is worth mentioning that the authenticity of the cDNA cloning results depends on the correctness of the primers defining the beginning and the end of the sequence. Usually, there are no problems with establishing the initial primer due to conserveness of the signal peptide of the whole prepropeptide. However, establishing the end of the AMP sequence is rather challenging and often leads to the wrong sequences [26,27]. Until recently, certain advantages of cloning involved unambiguous distinguishing between the isomeric Leu/Ile residues. Nevertheless, modern mass spectrometers enable resolving that problem with an EThcD MS^3^ experiment demonstrating characteristic losses from the side groups of these amino acids [28,29,30]. The efficiency of that approach is a bit lower than in the case of cDNA cloning, being limited by the structural ability to form intermediate z-ions with an N-terminal Leu/Ile [29].

The present study aimed to establish the skin peptidome of the Agile frog (Leap frog, Spring frog, Dalmatina frog, *Rana dalmatina*, Fitzinger, 1838 frog). It is also known as: *Rana agilis* Thomas, 1855; *Rana gracilis* Fatio, 1862; *Rana agiloides* Brunner, 1951; and *Rana mülleri* Brunner, 1959 [31]. Brown frog *Rana dalmatina* belongs to the *Rana* genus (true frog) containing 446 species by 2023 [31]. Its habitat stretches from southern Sweden to the north-east of Spain. It meets in Carpathian Ukraine, Sicily, the southern Balkans up to Greece, and north-east of the Asian part of Turkey [32]. It is widely distributed in Slovenia [33,34]. A certain peculiarity of that frog involves long hind legs. Being stretched out, they significantly exceed the length of the frog torso. The breeding takes place in creeks while the animals lead a terrestrial lifestyle and may be found up to 1700 m above the sea level [35,36]. The habitat of this species are glades and open sites within light deciduous woodland, with or without herbaceous vegetation, and less frequent in meadows and thickets. Outside the mating season, they can be found in very dry parts of the forest. It spawns in small wetlands within forests and at their edges [35,36]. This species is threatened by the drainage and eutrophication of breeding sites, destruction of suitable habitat, and replacement of deciduous forest habitat with unsuitable coniferous species. It is locally threatened by road mortality during breeding migrations [36,37].

Although the *Rana dalmatina* species was described a long time ago, its genome remains unknown, while its skin peptidome has been poorly studied so far. Only one 17-mer disulfide brevinin 1Da is known, being isolated by extraction from the flayed skin in 2004 [38]. Brevinin 1Da demonstrated high activity against *St*. *aureus* (MIC 7 μM) and moderate activity against *E. coli* (MIC 30 μM) [38]. It was reported that the methanol extracts of the flayed skin of *Rana dalmatina* and *Rana latastei* frogs may contain negligible levels of bradykinin-related peptides (BRPs) [39,40]. In the present study, we paid rather significant attention to this class of peptides, as bradykinin itself and its numerous analogs are found in the secretions of the majority of amphibian species [41]. These molecules play an important role in the defense against predators, in the transmission of signals of numerous reactions, including cardiovascular regulation and the stimulation of cell proliferation, contraction of gastric smooth muscle, hyperanalgesia, etc. [9,41]. The transcriptome of *Rana dalmatina* of the Croatian population obtained by cDNA cloning was reported in [27,42]. However, no comparison was provided with the actual secreted skin peptidome of that species. 

Continuing our research on the sequencing of skin peptides of various frog species, in the present work *Rana dalmatina* secretions of the Central Slovenian population were studied. The sequencing was performed by the manual interpretation of the tandem mass spectra (CID, HCD and EThcD), recorded with Orbitrap instruments.

## 2. Results and Discussion

Peptides discovered in the skin secretions of the Central Slovenian *R*. *dalmatina* samples are listed in Table 1. The table contains experimental and theoretical monoisotopic molecular masses, mass errors in *ppm*, peptide sequence and the reference first mentioning that peptide. Peptides reported for the first time are marked with an asterisk. The underlined portion of the sequence represents the S-S cycle. Identified isomeric Leu/Ile in the sequence of novel peptides are marked with bold. Identification codes for the peptides from the protein database UniProtKB/swiss-prot available at the BLAST platform are mentioned together with the peptides’ sequences.

All peptides were sequenced manually. The correctness of the sequences was confirmed using the Protein Calculator application by Thermo Fischer Scientific (Build 4.0.17) for the prediction of fragment ion masses in silico. Two examples are presented in the corresponding tables of predicted fragment ions for Brevinin 1Db and Temporin 1Da in the Supplementary Section. MS/MS data for all the experiments are available on a free repository. The reference is in the Supplementary Material. It should be mentioned once again that the genome of that frog remains unknown, while the composition of its skin peptidome is reported here for the first time.

Since our latest works were aimed at less abundant peptides, a change in the chromatographic conditions of our experiments was required. It led to a dramatic increase in the spectra number (up to 20,000 per chromatogram) and desirable use of commercial software. The latest versions of Peaks Studio using deep learning algorithms [43,44] demonstrated quite reasonable results for short linear peptides, but not for intact disulfide bond-containing ones [24]. In this study, Novor.Cloud was applied. It is a free online tool developed for de novo sequencing [45,46]. Novor showed results quite similar to Peaks Studio. It is an efficient tool for linear peptides, especially those producing quite intense and clear ion series, e.g., acidic spacers or Bradykinins containing N-terminal Arg.

The following peptide families were present in the secretions: brevinins 1, brevinins 2, melittin-related peptides (MRPs), temporins and BRPs. The obtained set of peptide families is quite similar to that of *R*. *temporaria,* belonging to the same species group of Dubois (1992) [47]. The absence of ranatuerins also confirms that similarity.

**Table 1:** Peptides identified in the skin secretion of Central Slovenian Brown frog *Rana dalmatina*.

**No.**	Peptides	Exp. MM, Da	Theor MM, Da	Δm, ppm	Sequences and ID Numbers	Refs
1	Brevinin 1Da	1809.096	1809.098	1.3	IIPLLLGKVVCAITKKC-OH	[38]
2 *	Brevinin 1Db	2607.472	2607.468	1.4	FFPAF**L**KVAAKVVPS**I**LCSITKKC-OH	
3 *	Brevinin 2D	3357.852	3357.855	0.9	GLLSGLKKVGKVVAKNVAVSLMDSLKCKISGDC-OH	
4	Brevinin 2Rd	3011.626	3011.626	0.3	GILDSLKNLAKNAAQILLNKASCKLSGQC-OH-P86025.1	[48]
5	FQ-22 (MRP)	2310.383	2310.383	0.3	FIGSALKVLAGVLPSVISWVKQ-NH_2_-P56924	[49]
6	Temporin A	1395.896	1395.897	0.4	FLPLIGRVLSGIL-NH_2_-P56917.2	[49]
7	Temporin B	1390.925	1390.928	1.8	LLPIVGNLLKSLL-NH_2_-P79874	[49]
8	Temporin C	1360.877	1360.881	2.6	LLPILGNLLNGLL-NH_2_-P56918	[49]
9	Temporin D	1376.880	1376.876	3.3	LLPIVGNLLNSLL-NH_2_-P56919	[49]
10	Temporin F	1367.887	1367.890	2.5	FLPLIGKVLSGIL-NH_2_-P56921	[49]
11	Temporin G	1456.890	1456.892	1.3	FFPVIGRILNGIL-NH_2_-P79875	[49]
12	Temporin H	1095.701	1095.702	0.5	LSPNLLKSLL-NH_2_-P79876	[49]
13	Temporin K	1121.754	1121.754	0.3	LLPNLLKSLL-NH_2_-P56923	[49]
14	Temporin M	1452.954	1452.954	0.3	FLPILGKVLSRVL-NH_2_	[50]
15 *	Temporin 1Da	1399.896	1399.896	0.3	F**L**P**LI**AG**LL**GKLF-NH_2_	
16	Bradykinin	1059.561	1059.561	0.4	RPPGFSPFR-OH-P0DM76.1	
17	[*des*Arg^9^]Br	903.460	903.460	0.3	RPPGFSPF-OH-P86628.1	
18	Br 1-7	756.390	756.392	2.5	RPPGFSP-OH	
19	Br 5-9	652.334	652.333	1.1	FSPFR-OH	
20	Br RI-5-10	765.416	765.417	1.8	FSPFRI-OH	
21	Br RI-10	1172.642	1172.645	3.0	RPPGFSPFRI-OH	
22	Br RA-1-11	1243.679	1243.683	2.9	RPPGFSPFRIA-OH	
23	[*des*Arg^1^]Br RA-2-11	1087.579	1087.581	2.3	PPGFSPFRIA-OH	
24	Br DR-11	1273.653	1273.657	2.9	DVRPPGFSPFR-OH	
25	[Thr^6^]Br	1073.572	1073.577	4.7	RPPGFTPFR-OH-C0HKA8.1	
26	[*des*Arg^9^][Thr^6^]Br	917.472	917.476	4.3	RPPGFTPF-OH	
27	[Thr^6^]Br 1-7	770.405	770.408	3.3	RPPGFTP-OH	
28	[Thr^6^]Br 5-9	666.347	666.349	2.9	FTPFR-OH	

**Figure 1:**
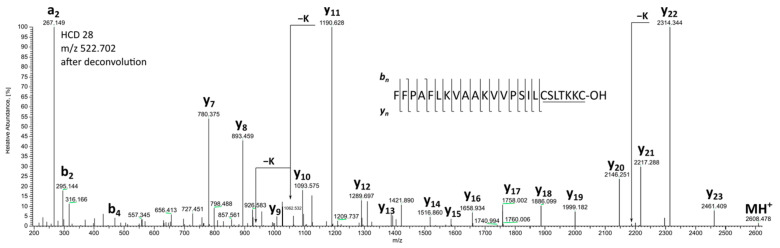
HCD spectrum of pentaprotonated brevinine 1Db (ion at *m*/*z* 522.702).

Four disulfide-containing peptides were identified in the secretions of the Central Slovenian agile frogs. Besides the earlier-reported brevinin 1Da [38], 24-mer brevinin 1Db was discovered. The peptides’ names were given according to the existing nowadays nomenclature of the frog peptides’ families [51]. Figure 1 presents the HCD spectrum of pentaprotonated brevinin 1Db (ion of *m/z* 522.702).

Partial sequence (TKKC) inside the intact C-terminal disulfide cycle was established using HCD and EThcD MS/MS tools. The losses of K and KK shown in Figure 1 from y_22_ and y_11_ ions, respectively, demonstrate secondary fragmentation after the cleavage of the C-terminal peptide bond in the primary y-ions [52].

**Figure 2:**
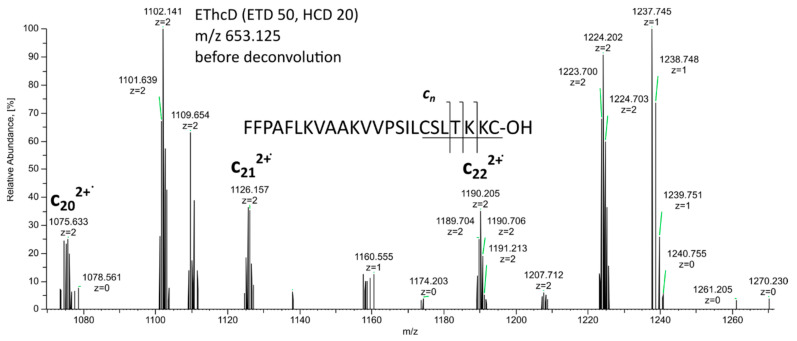
Selected part of the EThcD spectrum of tetraprotonated brevinine 1Db (*m*/*z* 653.121) corresponding to Rana box fragmentation.

The selected part of the EThcD MS^3^ spectrum shown in Figure 2 demonstrates crucial ions of the c-series, revealing the mentioned TKKC C-terminal sequence [22].

The search in the UniProtKB database [53] revealed certain sequence similarity of the linear part of brevinin 1Db with brevinin 1R from *Pelophylax ridibundus* (11 coincidences out of 17 aa) [48], while its Rana box completely matched the Rana box of brevinin 1AUa and 1AUb of brown frog *R*. *aurora aurora*: CSITKKC [9] by the accurate mass of the *y*_7_ ion and C-terminal sequence (TKKC) established with HCD and EThcD. 

**Figure 3:**
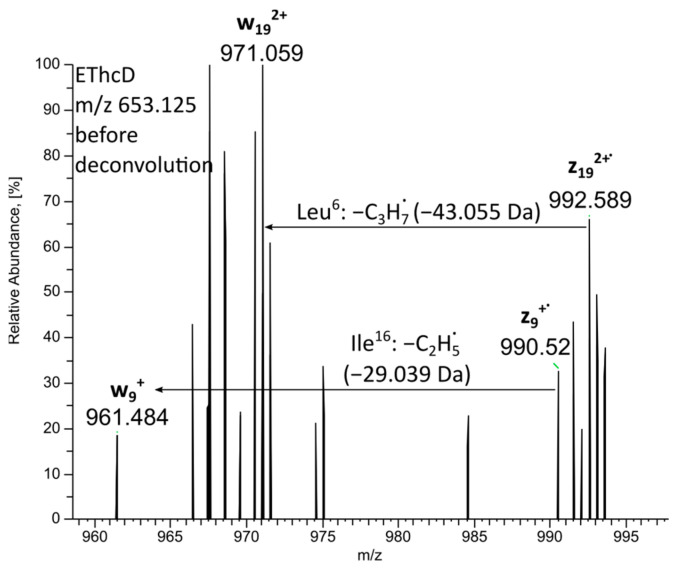
Identification of Leu^6^ and Ile^16^ in brevinin 1Db using EThcD (ETD 50, HCD 40) spectrum of *m*/*z* 653.125 ion.

Since we could not differentiate Leu/Ile in this case with MS/MS, the only doubt in the sequence involved position 20. Was there Leu or Ile? Two brevinins from *R*. *aurora aurora* definitely testified in favor of Ile. Moreover, in the quite rare 24-mer brevinins 1 [9], position 20 is conserved and always occupied by Ile (never Leu). Therefore, the final sequence of brevinine 1Db was defined as FFPAF**L**KVAAKVVPS**I**LCSITKKC-OH. The highlighted Leu^6^ and Ile^16^ in its sequence were established with the EThcD tool [19] (Figure 3).

Secretion of *Rana dalmatina* contains 33-mer brevinine 2D (Mm 3357.852 Da), with His^12^→Val^12^ being the only substitution relative to brevinin 2T in *R*. *temporaria* peptidome. 

Rather surprising was the presence of brevinine 2Rd in all samples from the Slovenian *R*. *dalmatina* species. It is a 29-mer peptide identical to that found in the secretion of the frogs from *Pelophylax esculentus complex* [48]. This fact was supported by the presence of the green frogs’ brevinins 1 and 2 sequences in the transcriptome of the Croatian population of *R*. *dalmatina* [27,42]. Table 2 is based on the data from the Croatian population of *R*. *dalmatina* [27] and serves herein for comparison. Table 2 also contains the name of the peptide structurally similar (all substitutions are mentioned in the last column) or identical to the sequence obtained by cloning, the amphibian species name from which it was isolated and the reference to the original paper. Brevinins marked rdal_38_11/1-23, rdal_103_32/1-24 and rdal_15_33/1-34 in Table 2 were earlier discovered in the secretions of species forming *Pelophylax esculentus complex* [6,48].

**Figure 4:**
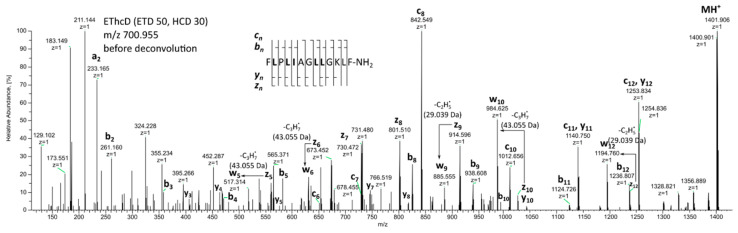
EThcD spectrum of doubly protonated Temporin 1Da (*m*/*z* 700.995) before deconvolution.

Besides the four mentioned disulfide-containing brevinins 1 and 2, secretion of *R*. *dalmatina* contained ten temporins as well as melittin-related peptide FQ-22 identical to that from *R. temporaria* [49]. Temporins represent a perspective family of antimicrobial peptides due to their short chains and broad spectrum of activities. They are studied better than other peptide families [58,59,60]. Eight of the identified temporins (A, B, C, D, F, K, G, H) were reported earlier by the M. Simmaco group, being discovered in European *R*. *temporaria* [49]. Temporin M was first detected in the secretion of the Moscow population of the same species [50]. Novel temporin 1Da was sequenced in the present work for the first time. The EThcD spectrum of that peptide (monoisotopic MM 1399.896 Da) reveals the full sequence as presented in Figure 4. Distinguishing between the isomeric Leu/Ile residues in the sequence of temporin 1 Da was conducted by the characteristic losses of isopropyl *vs* ethyl radicals from the side chains of z-ions in EThcD spectra [29,30]. This approach allowed us to identify five out of six isomeric residues (see w-ions in Figure 4). The search in the UniProtKB database did not reveal any known sequences for the discovered temporin 1 Da.

All three frogs of the Slovenian population of *R*. *dalmatina* contained several BRPs. Besides bradykinin itself, there were an agonist [*des*Arg^9^]BR of its receptor and its structural analog [Thr^6^]Br. Additionally, the products of bradykinin proteolysis (**18**, **19** in Table 1), C-extended forms of bradykinin (**21**, **22**), as well as their proteolytic forms (**20**, **22**), were detected. There was only one N-extended copy of bradykinin (**24**) and four proteolytic forms of bradykinin analog [Thr^6^]Br (**25**–**28**). The chromatographic peaks of the BRPs were low, as were the ion intensities of the protonated BRPs in the mass spectra, in all three samples. The targeted search was based on the characteristic *b*- and/or *y*-ions of bradykinin and its analog [Thr^6^]Br, as well as characteristic *b*- and/or *y*-ions of their extended copies [50].

The presence of the peptides predicted by cloning (Table 2) was also checked using targeted analysis. Ranacyclines are a family of short cyclic skin peptides with a conserved motive GCWTKSXXPKPC. They demonstrate a wide spectrum of antimicrobial properties, being also protease inhibitors [9]. However, the predicted by cloning ranacycline GALRGCWTKSIPPKPCKGK (rdal_108_19/1-19, Table 2, [27]) was not detected. By the way, its sequence is similar to ranacycline T of the related frog *R*. *temporaria*, with only one substitution: Ile^11^→Tyr^11^ [57].

A targeted analysis gave negative results also in the case of the peptide AAKIILNPKFRCKAAFC (rdal-58-29/1-17; Table 2) that obtained the name ranatuerin 2Ra. Together with the related ranatuerin R (AVNIPFKVKFR(CKAAFC)-OH), ranatuerin 2Ra was found by our group in the skin secretion of *P*. *ridibundus* of the Moscow and Central Slovenian populations, as well as in all three species of green frogs forming *P*. *esculentus complex* [48]. The distinguishing of the isomeric Leu/Ile residues in these peptides was carried out using tandem mass spectrometry [29,30]. These peptides also possess, besides antibacterial, protease inhibitory activity. The latter is possible due to the disulfide cycle (CKAAFC) in their sequence [9]. Later, Chen et al. [61], using noninvasive “shotgun” cDNA cloning, obtained the same sequences from the skin secretions of the European *P*. *esculentus* and Chinese *O. schmackeri,* with the only substitution in ranatuerin 2R (Lys^9^—His^9^ for *O. Schmackeri*), and assigned them to the kunitzin family with the new names: kunitzin-RE and kunitzin-OS, correspondingly. Besides antimicrobial activity against *E*. *coli* and weak hemolytic activity, kunitzins appeared to be potent inhibitors of trypsin [61]. Being conjugated with cationic cell-penetrating peptides, kunitzin-like trypsin inhibitors demonstrate a dramatic increase of their antibacterial and antitumor activities. That fact gives a chance to use these compounds as powerful antimicrobial and antitumor agents in the future [62].

A related issue worth a special mention involves the fact that intact antimicrobial peptides were hardly traceable, while their full sequences were often established by combining information from overlapping *C*- and *N*-terminal pieces. This fact significantly distinguishes those frogs from all Ranidae frogs studied earlier by our group. Most probably precisely the absence of the known nowadays protease inhibitors in the secretion of *R*. *dalmatina* brought to the fast degradation of almost all the antimicrobial peptides [6,8].

Taken for the comparison, transcriptome of the Croatian *R. dalmatina* frogs was obtained by parallel simultaneous identification of antimicrobial peptides from several anuran species using targeted DNA sequencing [27,42]. It contains many more peptides than can be detected studying skin peptidome directly, as not all potentially available peptides are secreted by amphibians [23,24,26,27,42]. However, transcriptome provides the sequences of peptides which were secreted by that particular frog species sometime in the past in the process of their distribution over the planet [63,64]. Facing a new habitat, amphibians start using novel, more efficient peptides of the same families with substitutions of some amino acids in the chain. This process is possible by mutation or gene duplication [63,64,65,66,67]. Thus, the skin secretion of a newly studied population of the same species may demonstrate peptides predicted by cDNA cloning a long time ago but never registered so far in the secretions [24]. Therefore, secreted antimicrobial peptides (AMPs) are the peptides that were selected by the frogs of each particular population from the whole array of AMPs genetically encoded in their chromosomes as the most efficient peptides to fight the surrounding pathogenic microbiota in the new habitat.

It is worth mentioning that even considering inter-populational differences between the Slovenian and Croatian species, we expected more similarities in their sequences. Actually, the only common peptide reported in both studies was brevinine 1Da. The reason for this result may involve the following issue. The success of cDNA cloning notably depends on the available information on the skin secretions of the species used in the study, as it helps correctly defining the primers to read genetically encoded sequences of the peptides. However, the skin peptidome of *R. dalmatina* was not studied so far. This issue might result in certain mistakes in obtaining the sequence and lead to certain discrepancy between its transcriptome (Table 2) and peptidome (Table 1).

## 3. Materials and Methods

### 3.1. Reagents

The following reagents were used: acetonitrile and methanol, HPLC gradient grade, Sigma-Aldrich (St. Louis, MO, USA); formic acid, HPLC gradient grade, Fluca (Buchs, Switzerland). Water was prepared by the MilliQ water purification system (Millipore, Billerica, MA, USA).

### 3.2. Skin Secretions

Three frogs of *Rana dalmatina* were caught in May 2021 and May 2022 in the Vrhnika suburb of Ljubljana, Slovenia. Skin secretions were obtained by mild electrical stimulation as described in [68,69]. We consider this method as the most efficient and appropriate for the purpose of the study. In brief, the moistened back of an animal was treated for 30–40 s depending on the animal size with a bipolar electrode of a laboratory electrostimulator (ESL-1): frequency, 50 Hz; voltage, 10 V; pulse duration, 5 ms. The secretion was washed out with 25 mL MilliQ water into container with the same volume of methanol [70]. After 15 min in a centrifuge at 3000 rpm, the supernatant was purified with a Millex-FH membrane PTFE 0.45 μm filter (Millipore, Billirica, MA, USA), concentrated at 35 °C with the rotary evaporator up to 1 mL and lyophilized. The samples were stored at −26 °C. All frogs were returned immediately into the place of their capture being alive. To inhibit proteases, methanol may be substituted by various organic or inorganic acids [71,72,73]. Another variant involves the immediate freezing of the fresh secretion in liquid nitrogen [74] or injection of noradrenaline/norepinephrine into the lymphatic sacs [75,76,77].

### 3.3. Mass Spectrometry

Mass spectra were obtained using the LC-MS/MS system with Easy nano-LC 1000 (Thermo Scientific, New York, NY, USA) chromatograph attached to an Orbitrap Elite ETD (Thermo Scientific, Germany) mass spectrometer. The chromatographic nano column (75 μm × 150 mm) made in the laboratory with stationary phase Aeris 3.6 μm WIDEPORE XB-C18 (Phenomenex, Torrance, CA, USA) was used. Dried secretion samples were re-suspended in 1% formic acid and 4% acetonitrile solution in water and injected in 2 μL aliquots. Solution A—0.1% formic acid in MilliQ water, B—80% of acetonitrile and 20% of 0.1% formic acid in MilliQ water. The separation involved the gradient of B from 5% to 60% in 120 min with an eluent flow rate of 250 nL/min. Resolving power of the mass spectrometer for m/z 400 was 240,000 in full MS mode and 60,000 in MS/MS mode. The HCD and CID tandem mass spectra were registered repetitively in automatic mode. The experimental details were as follows: inlet capillary temperature—200 °C, inlet capillary voltage—1.6 kV, normalized cell energy (NCE) in CID and HCD mode was 35 and 28, correspondingly.

EThcD spectra were obtained using the Orbitrap Fusion mass spectrometer (ThermoFisher Scientific, New York, NY, USA) and Easy nano-LC 1000 chromatograph. LC-separation was conducted at 35 °C with a 50 cm long reversed phase EASY spray nano column (PepMap, C18, 2 mm, 100 Å). The chromatographic separation involved a gradient solvent system containing (A) water with 2% acetonitrile and 0.1% formic acid and (B) acetonitrile with 2% water and 0.1% formic acid. The gradient was set up as follows: 4% (B) in 7 min, 4–50% (B) in 21 min, 50–80% (B) in 5 min, 80% (B) for 5 min 95% (B) and 95–4% (B) for 5 min. The flow rate was 300 nL/min. The mass spectrometer was operating in the data-dependent acquisition mode. A survey mass spectrum was acquired in the range of *m*/*z* 300–1700 with a nominal resolving power of 120,000 (AGC target of 8.0e5 with a maximum injection time of 50 ms). The selection of precursor ions was performed in the “top speed” mode of the charge states from 2 to 7, with the most intense precursor priority and minimal intensity of 50,000. Dynamic exclusion was disabled. MS/MS was performed with resolving power 15,000, AGC target 5.0e4, activation time 100 ms, maximum injection time 200 ms and collision energy with steps 10%, 20%, 30% and 40%. Fluoranthene was used in the ETD mode. The sequencing was carried out by manual tandem mass spectra interpretation. Isomeric Leu/Ile residues were distinguished in the EThcD spectra as reported in [29,30].

A targeted search was applied to detect certain N- and C-extended bradykinins. In that case, y- and b-ions characteristic for bradykinins and its extended copies were used as described in [50].

Additional MS/MS experimental information may be found in the Supplementary Material section.

### 3.4. Application of BLAST (Basic Local Alignment Search Tool)

The uniqueness of the primary structures of the de novo sequenced peptides in the secretion of *Rana dalmatina* was confirmed by the search of the similar structures in the UniProtKB/Swiss-Prot database, available on the electronic resource BLAST. The primary goal of the search involved the finding of the full identity of the sequenced peptide with that in the protein database Swiss-Prot. The finding would signify that the obtained sequence was established earlier and possesses an identification code. If the sequence was not found, the established experimental sequences were treated as novel primary structures.

## 4. Conclusions

*Rana dalmatina* demonstrated certain similarities with the related *R. temporaria* by the array of peptide families in their secretions. However, there is little similarity in the structures of the disulfide-containing peptide families, namely brevinins 1 and 2. The number of temporins secreted by *R. dalmatina* is significantly lower than discovered in the related *R. temporaria* from the Central Slovenian, Moscow and Arkhangelsk populations [22]. 

Similarly, the array of BRPs of *R. dalmatina* is notably poorer in comparison with that of *R. temporaria*, with bradykinin being the major peptide in the secretion [41]. *R. dalmatina* concedes to *R. temporaria* both in the assortment of BRPs (including C- and N-extended copies) and in the intensity of their chromatographic peaks. On the other hand, ranatuerins 2 were not detected in both frog species, although they are present in the secretions of other species of the group of Dubois (1992): *R. latastei* and *R. dubowski* [47]. 

Fast degradation of the antimicrobial peptides in the secretions of *R. dalmatina* may proceed probably due to the absence of the known nowadays frogs’ protease inhibitors and high bioactivity of the discovered brevinin 1Db, brevinin 2D and temporin 1Da as well as the corresponding necessity to deactivate them as soon as possible in order not to damage the frog itself [6,8].

It is worth special mentioning that the array of the applied tandem mass spectrometry tools enabled revealing the whole sequences of all detected peptides, including differentiation between isomeric Leu/Ile, and the portion inside the disulfide cycle. There were no chemical derivatization steps applied, while the method may be treated as top-down de novo MS sequencing.

## Figures and Tables

**Table 2:** Transcriptome of Croatian *Rana dalmatina* obtained by cDNA cloning [27].

No.	Peptide Code	cDNA Cloning Sequence	Reference *
Group 1—Shot linear **			
1	rdal_51_25/1-15	FLPVIAGVLSKLFGK	Temp 1Da; *R. dalmat.* V^4^→L^4^; V^8^→L^8^ S^10^→G^10^
2	rdal_21_605/1-6	ILPLIGKVLSGILAK	Temp F; *R. temporar*. I^1^→F^1^ [49]
3	rdal_44_43/1-15	LLPIVGNLLNDLLGK	Temp D, *R. temp* Asp^11^→Ser^11^ [49] Temp AV *R. arv* [54]
4	rdal_226_12/1-14	FLPIVTNLLLRFVG	
5	rdal-8-600/1-18	FLGFVGQALNALLGKLGK	
**Group 2—Shot linear ranabox**			
6	rdal-38-11/1-23	FFPAIFRLVAKVVPSIICSVTKN	Brev 1R, *P. ridibundus* CSVTKKC [49]
7	rdal_103_32/1-24	FLPLLAGLAANFLPKIFCKITRKC	Brev 1E, *P. escul* [55]
8	rdal-3-1108/1-20	IVPILLGVVPQLVCAITKKC	Brev1Ita, *R. italica* I^4^→F^4^; V^8^→M^8^ Q^11^→K^11^; A^15^→L^15^ [56]
9	rdal-53-31/1-17	IIPLLLGKVVCAITKKC	Brev 1Da, *R. dalmat* [38]
**Group 3—Brevinin helical ranabox**			
10	rdal_15_33/1-34	GILLDKLKNFAKTAGKGVLQSLLNTASCKISGQC	Brev 2Ec, *R. escul* [6]
**Group 4—Esculentin helical ranabox**			
11	rdal-6-971/1-31	FLWETVKNFGKTFTLNILDKLKCKIGGECPP	Brev 2Tc, *R. tempor* V^6^→I^6^; T^12^→K^12^; D^19^→H^19^; E^28^→G^28^ [6]
12	rdal_120_5/1-37	GILSLVKGIAKLAGKGLAKEGGKFGLELMACKIAKQC	Escul 2a; *P. escul.* I^9^→V^9^; I^29^→M^29^ [55]
**Group 5—likely beta hairpin**			
13	rdal_108_19/1-19	GALRGCWTKSIPPKPCKGK	Ranacyc T; *R tempor* I^11^→ Y^11^ [57]
14	rdal-32-284/1-24	VPQLCFKFQKVIYCEINKTLPNFA	
**Group 6—Kunetzin**			
15	rdal-58-29/1-17	AAKIILNPKFRCKAAFC	Ranat 2Ra, *P. ridib* [48]
**Group 7 Pro-rich predicted by cloning** No peptides in that group			
**Group 8 Undefined**			
16	rdal-77-6/1-35	FLPLVLGKTHSFSEQAELLSWKSSNVEYHLPKCTDV	
17	rdal_7_282/1-9	GIVEAWPLR	
18	rdal_40_14/1-15	GLEVLGKILSGLLGK	Temp 1AVa; *R. arv* [54]
19	rdal-39-24/1-32	NLLGFLQGAKDILKECEADNYQGWLCESSYKPQ	
20	rdal-110-77/1-21	LANRAARNTQNVLNAITCTL	

* Reference column is added by us. ** Division by groups is kept as in [27].

## Data Availability

The data presented in this study are available for a limited time on request from the corresponding author.

## Supplementary Materials

The following supporting information can be downloaded at: https://www.mdpi.com/article/10.3390/molecules28207118/s1, Figure S1. Electrostimulator for the “milking procedure”; Figure S2. Rana dalmatina “milking” process; Figure S3. Brevinin 1Db mass spectra; Table S1: Brevinin 1Db in silico fragmentation (Protein Calculator application by Thermo Fischer Scientific (Build 4.0.17)); Table S2. Temporin 1Da in silico fragmentation (Protein Calculator application by Thermo Fischer Scientific (Build 4.0.17)). The raw files of all experimental data may be accessed by the following link https://disk.yandex.ru/d/C511Ho8TMtLcVQ; Orbitrap Fusion Method Summary; The results of automatic search engine Novor.cloud may be found using the following link: https://app.novor.cloud/analyses/0b5c881a-c70c-4f11-9197-c0fe92ccb0a6?accessToken=eyJhbGciOiJIUzI1NiJ9.eyJzdWIiOiIwYjVjODgxYS1jNzBjLTRmMTEtOTE5Ny1jMGZlOTJjY2IwYTYiLCJhY3QiOiJpZHZhc2lsaWV2YUBnbWFpbC5jb20ifQ.nJ4iwesp2naovLPk9yRdTS5_2wNi5zWtV9TyheHG5RM.

## Sample Availability

Samples of the secretion were completely used up during the research.
