# Supplementary material for: Tandem Mass Spectrometry de novo Sequencing of the Skin Defense Peptides of the Central Slovenian Agile Frog Rana dalmatina
Source: Molecules. 2023 Oct 16;28(20):7118. doi: 10.3390/molecules28207118 (PMC10608968; doi:10.3390/molecules28207118)
Supplement: Supplementary file 1 [file molecules-28-07118-s001.zip › molecules-2643018-supplementary.pdf]

Tandem Mass Spectrometry De Novo Sequencing of the Skin Defense Peptides of the Central Slovenian Agile Frog *Rana dalmatina*

Tatiana Yu. Samgina [1,2,*], Irina D. Vasileva [2], Polonca Trebše [3], Gregor Torkar [4], Alexey K. Surin [5], Zhaowei Meng [6], Roman A. Zubarev [6,7,8] and Albert T. Lebedev [1,2,*]

1 Department of Materials Science, MSU-BIT University, Shenzhen 517182, China
2 Department of Organic Chemistry, Lomonosov Moscow State University, 119991 Moscow, Russia;
idvasilieva@gmail.com
3 Faculty of Health Sciences, University of Ljubljana Zdravstvena Pot 5, 1000 Ljubljana, Slovenia; polonca.trebse@zf.uni-lj.si
4 Department for Biology, Chemistry and Home Economics, University of Ljubljana Faculty of Education, Kardeljeva ploščad 16, Ljubljana, Slovenia; gregor.torkar@pef.uni-lj.si
5 Pushchino Branch, Shemyakin–Ovchinnikov Institute of Bioorganic Chemistry, Russian Academy of Sciences, Prospekt Nauki 6, Pushchino, 142290 Moscow, Russia; alan@vega.protres.ru
6 Department of Medicinal Biochemistry and Biophysics, Division of Molecular Biometry, Karolinska Institutet, Stockholm, Sweden; zhaowei.meng@ki.se (Z.M.); roman.zubarev@ki.se (R.A.Z.)
7 The National Medical Research Center for Endocrinology, 115478 Moscow, Russia
8 Department of Pharmacological & Technological Chemistry, I.M. Sechenov First Moscow State Medical University, 119146 Moscow, Russia
* Correspondence: samguina@yandex.ru (T.Y.S.); mocehops@gmail.com (A.T.L.)

Supplementary information

The raw files of all experimental data may be accessed by the following link
https://disk.yandex.ru/d/C511Ho8TMtLcVQ

The results of automatic search engine Novor.cloud may be found using the following link:
https://app.novor.cloud/analyses/0b5c881a-c70c-4f11-9197-c0fe92ccb0a6?accessToken=eyJhbGciOiJIUzI1NiJ9.eyJzdWIiOiIwYjVjODgxYS1jNzBjLTRmMTEtOTE5Ny1jMGZlOTJjY2IwYTYiLCJhY3QiOiJpZHZhc2lsaWV2YUBnbWFpbC5jb20ifQ.nJ4iwesp2naovLPk9yRdTS5_2wNi5zWtV9TyheHG5RM

Table S1. Brevinin 1Db in silico fragmentation (Protein Calculator application by Thermo Fischer Scientific (Build 4.0.17))

Brevinin 1Db					
no	acid	B [M+H]+	Y [M+H]+	C° [M+H]+	C° [M+2H]2+
1	F	148.0757	-	164.0944	82.55085
2	F	295.1441	2461.407	-	-
3	P	392.1969	2314.339	408.2156	204.6114
4	A	463.234	2217.286	479.2527	240.13
5	F	610.3024	2146.249	626.3211	313.6642
6	L	723.3865	1999.181	739.4052	370.2062
7	K	851.4814	1886.097	867.5001	434.2537

8	V	950.5498	1758.002	966.5686	483.7879
9	A	1021.587	1658.933	1037.606	519.3065
10	A	1092.624	1587.896	1108.643	554.825
11	K	1220.719	1516.859	1236.738	618.8725
12	V	1319.787	1388.764	1335.806	668.4067
13	V	1418.856	1289.696	-	-
14	P	1515.909	1190.627	1531.927	766.4673
15	S	1602.941	1093.574	1618.959	809.9833
16	I	1716.025	1006.542	1732.043	866.5254
17	L	1829.109	893.4583	1845.127	923.0674
18	C	1931.11	780.3743	1947.129	974.0681
19	S	2018.142		2034.161	1017.584
20	I	2131.226		2147.245	1074.126
21	T	2232.274		2248.293	1124.65
22	K	2360.369		2376.388	1188.697
23	K	2488.464		2504.483	1252.745
24	C	-		-	-
sequence :FFPAFLKVAAKVVPSILCSITKKC					

Table S2. Temporin 1Da in silico fragmentation (Protein Calculator application by Thermo Fischer Scientific (Build 4.0.17))

Temporin 1Da						
no	acid	B [M+H]+	C [M+H]+	Y [M+H]+	W [M+H]+	Z° [M+H]+
1	F	148.0757	165.1022	-	-	-
2	L	261.1598	-	1253.834	1194.761	1237.816
3	P	358.2125	375.2391	1140.75	1097.708	-
4	L	471.2966	488.3231	1043.698	984.6241	1027.679
5	I	584.3806	601.4072	930.6135	885.555650/ 899.571300	914.5953
6	A	655.4178	672.4443	817.5294		801.5113
7	G	712.4392	729.4658	746.4923		730.4741
8	L	825.5233	842.5498	689.4709	630.3974	673.4527
9	L	938.6074	955.6339	576.3868	517.3133	560.3686
10	G	995.6288	1012.655	463.3027		447.2846
11	K	1123.724	1140.75	406.2813	332.1969	390.2631
12	L	1236.808	1253.834	278.1863	219.1128	262.1681
13	F	-	-	165.1022	72.04439	149.0841
sequence :FLPLIAGLLGKLF						

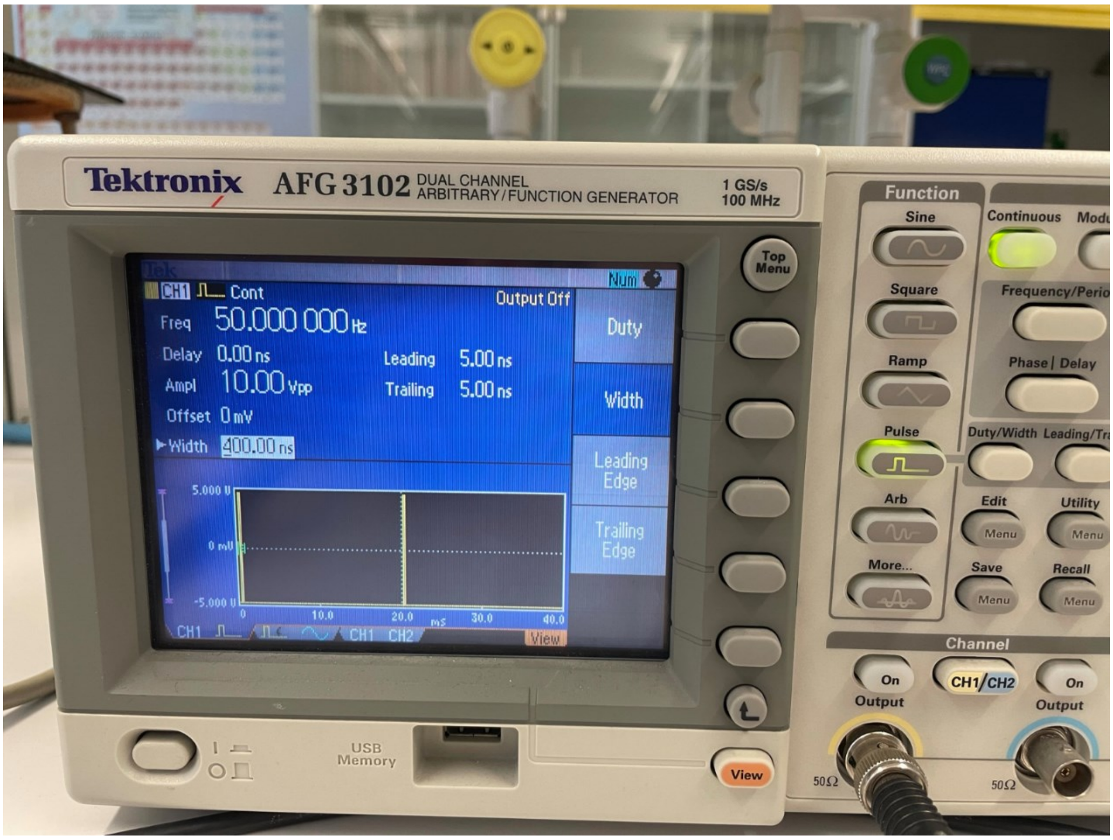

Figure S1. Electrostimulator for the “milking” procedure

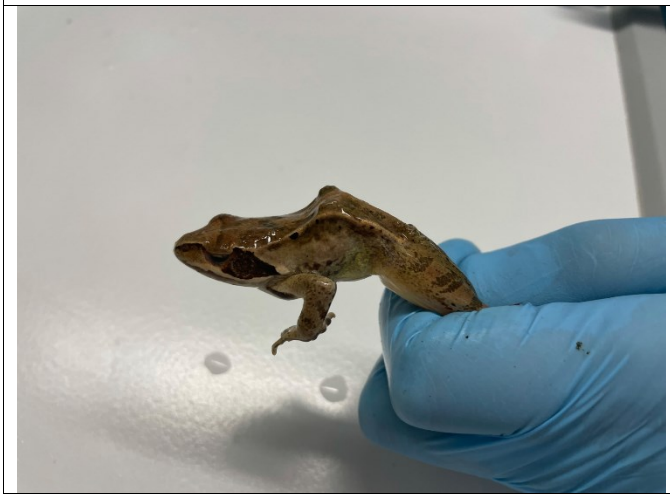

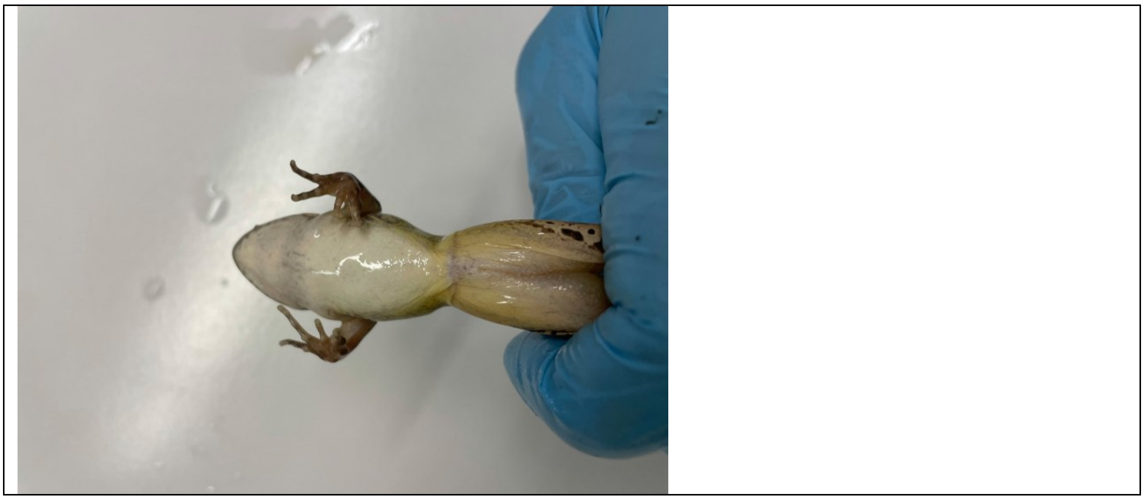

Figure S2. a,b. Rana dalmatina "milking" process

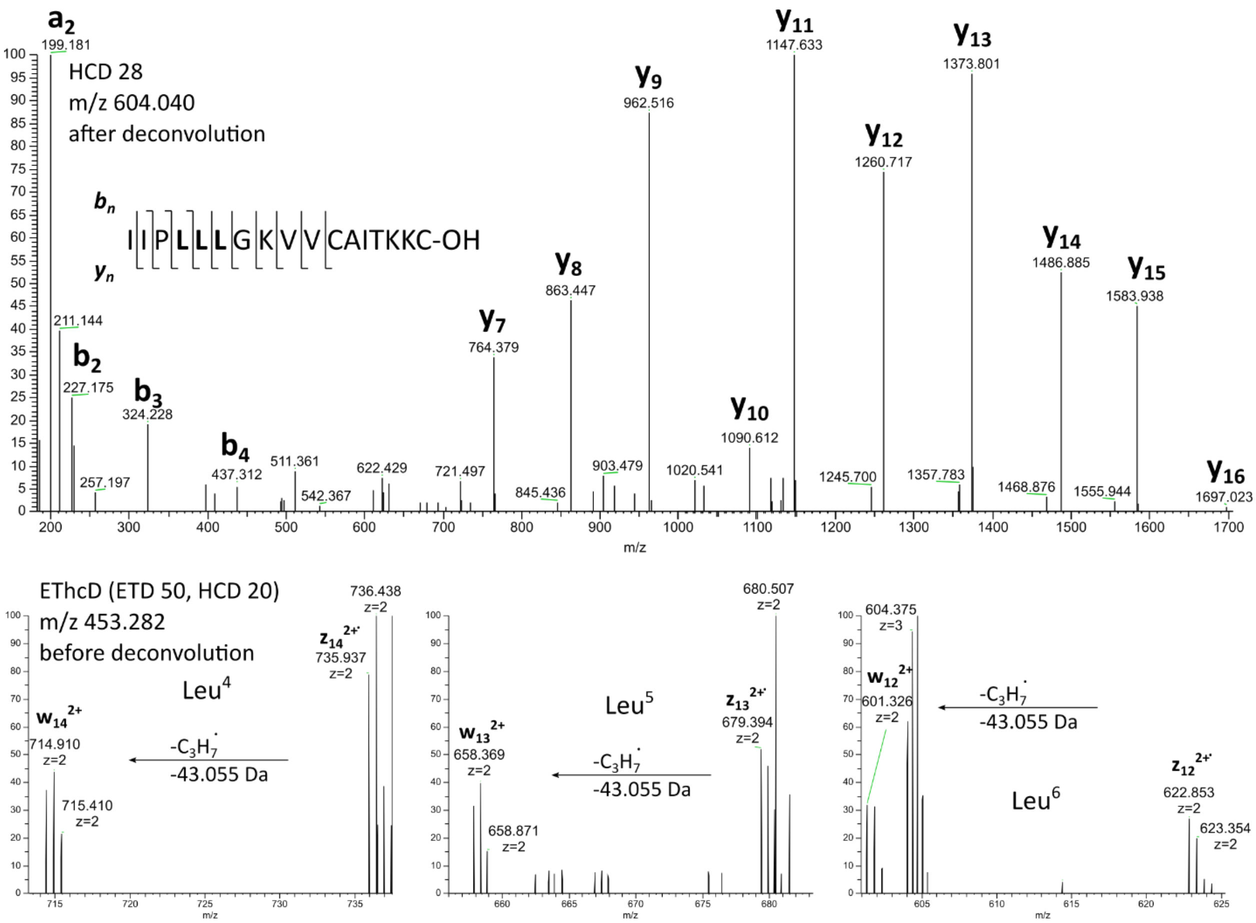

Figure S3. Brevinin 1Db mass spectra

Orbitrap Fusion Method Summary

Creator: 8R74CY1\Orbitrap Fusion Last Modified: 12/17/2021 12:32:19 by 8R74CY1\Orbitrap Fusion

Global Settings

- Use Ion Source Settings from Tune = False
- Method Duration (min)= 90
- Ion Source Type = NSI
- Spray Voltage: Positive Ion (V) = 1900
- Spray Voltage: Negative Ion (V) = 600
- Infusion Mode (LC)= False
- Sweep Gas (Arb) = 0
- Ion Transfer Tube Temp (°C) = 300
- APPI Lamp = Not in use
- FAIMS Mode = Not Installed
- Internal Mass Calibration= User Defined Lock Mass
- Application Mode = Peptide
- Pressure Mode = Standard
- Default Charge State = 4
- Advanced Peak Determination = True
- Xcalibur AcquireX enabled for method modifications = False
- Internal Cal Positive
  - m/z
  - 445.12003

Experiment 1

Experiment Name = Orbitrap CID-HCD-ETD
Start Time (min) = 0
End Time (min) = 90

Scan MasterScan

Desired minimum points across the peak = 6
MSn Level = 1
Use Wide Quad Isolation = True
Detector Type = Orbitrap
Orbitrap Resolution = 240K
Mass Range = Normal
Scan Range (m/z) = 300-2000
Maximum Injection Time (ms) = 100
AGC Target = 400000
Normalized AGC Target = 100%
Microscans = 1
Maximum Injection Time Type = Custom
RF Lens (%) = 60
Use ETD Internal Calibration = False
DataType = Profile
Polarity = Positive
Source Fragmentation = False
Scan Description =
Enhanced Resolution Mode = Off

Filter MIPS

Relax Restrictions when too few Precursors are Found = True
MIPS Mode = Peptide

Filter ChargeState

Include charge state(s) = 2-20
Include undetermined charge states = True

Filter DynamicExclusion

Exclude after n times = 1
Exclusion duration (s) = 5
Mass Tolerance = ppm
Mass tolerance low = 10
Mass tolerance high = 10
Use Common Settings = False
Exclude isotopes = True
Perform dependent scan on single charge state per precursor only = False
Exclude Within Cycle = False

Filter IntensityThreshold

Maximum Intensity = 1E+20
Minimum Intensity = 100000
Relative Intensity Threshold = 0
Intensity Filter Type = IntensityThreshold

Data Dependent Properties
Data Dependent Mode= Number of Scans
Number of Dependent Scans= 5

Scan Event 1

Scan ddMSnScan
MSn Level = 2
Desired minimum points across the peak = 6
Isolation Mode = Quadrupole
Isolation Window = 1.6
Isolation Offset = Off
Enable Auto PTR Windows = False
Reported Mass = Original Mass
Multi-notch Isolation = False
Scan Range Mode = Auto
Scan Priority= 1
Collision Energy Mode = Fixed
ActivationType = CID
Collision Energy (%) = 35
Activation Time (ms) = 10
Activation Q = 0.25
Multistage Activation = False
Detector Type = Orbitrap
Orbitrap Resolution = 60K
Maximum Injection Time (ms) = 200
AGC Target = 50000
Normalized AGC Target = 100%
Inject ions for all available parallelizable time = False
Microscans = 1
Maximum Injection Time Type = Custom
Use ETD Internal Calibration = False
DataType = Profile
Polarity = Positive
Source Fragmentation = False
Scan Description =
Enhanced Resolution Mode = Off

Scan Event 2

Scan ddMSnScan
Desired minimum points across the peak = 9
MSn Level = 2
Isolation Mode = Quadrupole
Isolation Offset = Off
Isolation Window = 1.6
Enable Auto PTR Windows = False
Reported Mass = Original Mass
Multi-notch Isolation = False
Scan Range Mode = Auto
Scan Priority= 1

ActivationType = HCD
Collision Energy Mode = Fixed
Collision Energy (%) = 28
Detector Type = Orbitrap
Orbitrap Resolution = 60K
Maximum Injection Time (ms) = 200
AGC Target = 50000
Inject ions for all available parallelizable time = False
Normalized AGC Target = 100%
Microscans = 1
Maximum Injection Time Type = Custom
Use ETD Internal Calibration = False
DataType = Profile
Polarity = Positive
Source Fragmentation = False
Scan Description =
Enhanced Resolution Mode = Off

Scan Event 3

Scan ddMSnScan

Desired minimum points across the peak = 9
MSn Level = 2
Isolation Mode = Quadrupole
Isolation Offset = Off
Isolation Window = 1.6
Enable Auto PTR Windows = False
Reported Mass = Original Mass
Multi-notch Isolation = False
Scan Range Mode = Auto
Scan Priority= 1
ActivationType = ETD
Use calibrated charge dependent ETD parameters = False
ETD Reaction Time (ms) = 50
ETD Reagent Target = 200000
Max ETD Reagent Injection Time (ms) = 200
ETD Supplemental Activation = False
Detector Type = Orbitrap
Orbitrap Resolution = 60K
Maximum Injection Time (ms) = 200
AGC Target = 50000
Inject ions for all available parallelizable time = False
Normalized AGC Target = 100%
Microscans = 1
Maximum Injection Time Type = Custom
Use ETD Internal Calibration = False
DataType = Profile
Polarity = Positive
Source Fragmentation = False
Scan Description =
Enhanced Resolution Mode = Off
